# Unveiling the Potential of Migrasomes: A Machine-Learning-Driven Signature for Diagnosing Acute Myocardial Infarction

**DOI:** 10.3390/biomedicines12071626

**Published:** 2024-07-22

**Authors:** Yihao Zhu, Yuxi Chen, Jiajin Xu, Yao Zu

**Affiliations:** 1 International Research Center for Marine Biosciences, Ministry of Science and Technology, Shanghai Ocean University, Shanghai 201306, China; 2 Key Laboratory of Exploration and Utilization of Aquatic Genetic Resources, Ministry of Education, Shanghai Ocean University, Shanghai 201306, China; 3 Department of Laboratory Medicine, School of Medicine, Jiangsu University, Zhenjiang 212013, China; 4 Marine Biomedical Science and Technology Innovation Platform of Lin-Gang Special Area, Shanghai 201306, China

**Keywords:** migrasome, acute myocardial infarction, bioinformatics, machine learning, diagnosis

## Abstract

Background: Recent studies have demonstrated that the migrasome, a newly functional extracellular vesicle, is potentially significant in the occurrence, progression, and diagnosis of cardiovascular diseases. Nonetheless, its diagnostic significance and biological mechanism in acute myocardial infarction (AMI) have yet to be fully explored. Methods: To remedy this gap, we employed an integrative machine learning (ML) framework composed of 113 ML combinations within five independent AMI cohorts to establish a predictive migrasome-related signature (MS). To further elucidate the biological mechanism underlying MS, we implemented single-cell RNA sequencing (scRNA-seq) of cardiac *Cd45*+ cells from AMI-induced mice. Ultimately, we conducted mendelian randomization (MR) and molecular docking to unveil the therapeutic effectiveness of MS. Results: MS demonstrated robust predictive performance and superior generalization, driven by the optimal combination of Stepglm and Lasso, on the expression of nine migrasome genes (*BMP1*, *ITGB1*, *NDST1*, *TSPAN1*, *TSPAN18*, *TSPAN2*, *TSPAN4*, *TSPAN7*, *TSPAN9*, and *WNT8A*). Notably, *ITGB1* was found to be predominantly expressed in cardiac macrophages in AMI-induced mice, mechanically regulating macrophage transformation between anti-inflammatory and pro-inflammatory. Furthermore, we showed a positive causality between genetic predisposition towards *ITGB1* expression and AMI risk, positioning it as a causative gene. Finally, we showed that ginsenoside Rh1, which interacts closely with ITGB1, could represent a novel therapeutic approach for repressing ITGB1. Conclusions: Our MS has implications in forecasting and curving AMI to inform future diagnostic and therapeutic strategies for AMI.

## 1. Introduction

Acute myocardial infarction (AMI) is the most severe form of cardiovascular disease, causing 7.4 million deaths annually [1]. AMI is typically caused by thrombus formation or vascular occlusion, characterized by a sudden decrease in blood flow to the myocardium, ultimately resulting in heart failure and death [2]. Over the past few decades, cardiac biomarkers have garnered widespread attention as indicators of AMI. Cardiac troponin (cTn) remains recognized as the “gold standard” for diagnosing AMI due to its high sensitivity, specificity, and short turnover time [3]. When myocardial cells are damaged, the presence of cTn in the bloodstream increases in direct proportion to the severity of the injury [4]. However, evidence suggests that the levels of cTn may increase in patients with heart failure, sepsis, and chronic kidney disease, leading to false-positive results [5]. Although substantial progress has been made, the diagnosis of AMI remains a complex challenge due to its multifactorial nature. Therefore, it is necessary to search for biomarkers with higher specificity for the early diagnosis of AMI.

The migrasome is a newly discovered type of extracellular vehicle (EV) located on retracting fibers (RFs) behind migrating cells, whose generation depends on cell migration [6]. Migrasomes contain various intracellular substances, such as chemokines, cytokines, and growth factors, which can be released into the extracellular space through migracytosis, thereby impacting the physiological and pathological processes of surrounding cells [7]. Increasing evidence indicates that the migrasome mediates diverse pathological processes alongside its role in organ morphogenesis, clearance of damaged mitochondria under mild mitochondrial stress, and facilitation of the lateral transfer of proteins and mRNAs [8,9,10]. Existing research has also demonstrated the crucial role of migrasomes in maintaining vascular homeostasis. TSPANs are crucial structural components in migrasomes, prevalent throughout the cardiovascular system [11]. TSPANs play integral roles in various physiological processes, including thrombosis, hemostasis, angiogenesis, vascular injury response, and cardiac development [12]. Notably, *TSPAN4* is abundantly expressed in migrasomes, serving as the most distinct marker for migrasomes [13]. It has been proven that the expression of *TSPAN4* is closely tied to atherosclerosis regression-related macrophages and intraplaque hemorrhage, which is also upregulated in AMI-induced murine models [14]. Hence, migrasomes may serve as promising candidate biomarkers for AMI.

**Figure 1:**
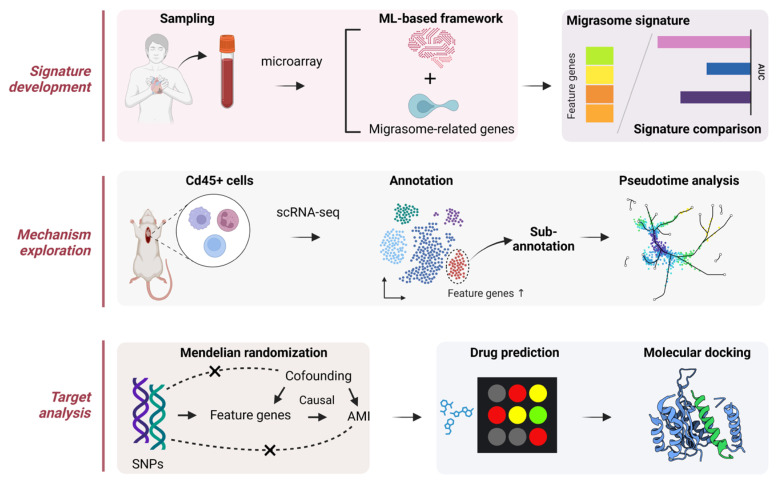
Schematic overview of the study. This study can be divided into three parts: signature development, mechanism exploration, and target analysis.

As noted above, there are many precedents for migrasomes being physiopathological indicators of cardiovascular diseases. To the best of our knowledge, limited studies have been performed on the diagnostic values of migrasomes. Therefore, the clinical application of migrasomes in diagnosing and assessing AMI remains obscure. Herein, we speculate on the great potential of migrasomes to predict the risk of AMI. A flowchart of our study is shown in Figure 1. Leveraging a machine learning (ML)-based computational framework, we initially developed a robust migrasome-related signature (MS) to predict patients with AMI accurately. Performing single-cell RNA sequencing (scRNA-seq) of cardiac *Cd45*+ cells isolated from murine models with induced AMI, we then investigated the cell-specific expression pattern of MS at single-cell resolution. Additionally, Mendelian randomization (MR) analysis allowed us to delve into the casual association between MS expression and the risk of AMI. Our results demonstrated that MS could serve as a promising diagnostic biomarker and druggability target for AMI.

## 2. Materials and Methods

### 2.1. Acquisition and Processing of Data

Gene expression profiling of peripherical blood (PB) derived from AMI patients was generated from the Gene Expression Omnibus (GEO) database [15]. Our study involves five cohorts of AMI and control cases: GSE123342, GSE29532, GSE60993, GSE61144, and GSE97320 [16,17,18,19]. Detailed information for these datasets is summarized in Table 1. The raw data were processed using the Robust Multi-Array Average (RMA) algorithm for background subtraction and data normalization [20]. We initially filtered out the samples with extreme and missing values in each cohort. Low-abundance genes with expression of less than 1 in all samples were deleted in each cohort. For genes detected by multiple probes, their average expression values were calculated. We performed quantile and log transformations to normalize the expression levels of genes in each cohort. We used GSE123342 as the training cohort for developing the migrasome-related signature (MS). GSE29532, GSE60993, GSE61144, and GSE97320 were selected as four independent testing cohorts to validate the predictive performance of MS. To deepen our understanding of the biological mechanisms of MS in the progression of AMI, we used GSE163465, a scRNA-seq dataset mapping the *Cd45*+ cells isolated from the hearts of AMI murine models [21].

### 2.2. Development and Validation of a Migrasome-Related Signature (MS)

Leveraging an integrative ML framework, we could identify the most predominant migrasome-related features for classification modeling between control and AMI cases, thereby establishing an MS model. We initially conducted a literature review and collected a list of migrasome-related genes (*n* = 35), including membrane markers (*n* = 17), protein markers (*n* = 4), and signaling molecules (*n* = 14). The list and literature review on migrasome-related genes can be found in Supplementary Table S1 and Supplementary File S1, respectively. Herein, we used GSE123342 (with the largest sample number) as the training cohort to develop an MS. The other four cohorts (GSE29532, GSE60993, GSE61144, and GSE97320) were selected as independent testing cohorts to assess the predictive power of MS. By overlapping the migrasome-related genes within the training and testing cohorts, we obtained a total of 23 migrasome-related genes as feature input: *BMP1*, *BMP2*, *BMP7*, *CXCL12*, *ITGA3*, *ITGA5*, *ITGB1*, *NDST1*, *PDGFD*, *PIGK*, *TSPAN1*, *TSPAN13*, *TSPAN18*, *TSPAN2*, *TSPAN3*, *TSPAN4*, *TSPAN5*, *TSPAN6*, *TSPAN7*, *TSPAN9*, *WNT11*, *WNT5B*, and *WNT8A*. The detailed procedure for establishing MS was as follows: (1) Before constructing an MS, we initially performed a Z-score transformation to normalize the gene expressions, which guaranteed comparability between different cohorts and increased the running speed. (2) Discovery of the MS was performed in the training cohort (GSE123342). To develop an MS, we established an ML-based integrative framework. Ten ML algorithms, including least absolute shrinkage and selection operator (Lasso), ridge, elastic network (Enet), stepwise generalized linear model (Stepglm), support vector machine (SVM), generalized linear model by likelihood-based boosting (glmBoost), linear discriminant analysis (LDA), partial least squares regression for generalized linear models (plsRglm), random forest (RF), gradient boosting machine (GBM), eXtreme gradient boosting (XGBoost), and NaiveBayes, were applied [22,23]. Descriptions of these 12 ML algorithms are summarized in Supplementary File S2. In this computational framework, four algorithms capable of feature screening (Lasso, RF, Stepglm, and glmBoost) were benchmarked to select feature genes from the 23 migrasome-related genes. Furthermore, the other eight algorithms were used to fit models based on the feature genes. Altogether, 113 ML combinations were generated to tune hypermeters and fit models under 10-fold cross-validation (CV). (3) All these ML combinations were validated in the four independent testing cohorts. To assess the predictive performance of each combination, the area under the curve (AUC) value was calculated for each cohort. The ML combination with the highest average AUC within the training and testing cohorts was considered optimal. We called this optimal combination MS. The experimental setting for developing an MS, including the R package calls, cross-validation, and hyper-parameter optimization, can be found in Supplementary File S2.

### 2.3. Comparison of the MS and Other Published Signatures

Comparing the performance differences between our MS and other reported signatures would help us to evaluate whether our signature’s predictive performance was superior. We retrospectively conducted literature retrieval, which can be found in Supplementary File S1. To ensure equitable comparability, we collected reported mRNA signatures developed to predict AMI. We ultimately gathered a list of 63 published signatures that forecast AMI, which are summarized in Supplementary Table S2. Signatures with more than 30% of genes that were not matched in our cohorts were excluded. An AUC score was computed for each signature in the training and testing cohorts.

### 2.4. Pre-Processing of scRNA-Seq Data

From a single-cell resolution perspective, we could investigate the detailed expression pattern of our MS in certain cell populations during AMI progression. We used GSE163465, a scRNA-seq dataset that maps *Cd45*+ cells from murine models of AMI at different time points (Day 1, Day 3, Day 5, and Day 7) to investigate the expression patterns and dynamics of MS in the progression of AMI. The pre-processing procedure for scRNA-seq data includes data filtering, normalization, identification of highly variable genes, and dimensionality reduction, as shown in Supplementary Figure S2. First, we leveraged the R package “Seurat” (version 4.3.0) to perform quality control of the raw data [24]. Poor-quality cells with less than 200 gene counts (or larger than 5000 gene counts) and mitochondrial gene proportions larger than 20% were filtered out. Second, log transformation was conducted to normalize the gene expressions of the remaining cells. Third, we selected the top 2000 highly variable genes for downstream analysis. A linear transformation was subsequently executed to scale the data for the shift of expression and the removal of variance. Fourth, principal component analysis (PCA) was performed based on the top 2000 highly variable genes. The top 15 principal components (PCs) were chosen as the optimal dimensionality for t-distributed stochastic neighbor embedding (t-SNE).

### 2.5. Cell Type Annotation and Pseudo-Time Analysis

Performing cell type annotation, we investigated the cell composition in AMI progression and reported the cell-specific patterns of MS. Next, based on the top 15 PCs with the highest variance (resolution = 0.1), we leveraged the t-SNE approach to visualize the clustering of cells. To highlight the role of MS in immune cells, we removed clusters with low expression of *Cd45*. The identities of clusters were determined based on the known marker genes [21]. In sum, we annotated four cell populations: (1) Macrophage/Monocyte (*C1qa*, *C1qb*, *C1qc*, *Adgre1*, *Ly6c2*, *Ccr2*, *Csf1r*, and *Cd68*); (2) T/NK cell (*Trbc2*, *Cd3d*, *Cd3e*, *Cd3g*, *Cd4*, *Cd8b1*, *Klra8*, *Klrb1c*, *Klrc1*, *Ncr1*, *Klra4*, *Klrc2*); (3) Neutrophil (*S100a9*, *S100a8*, *Lcn2*, and *Cxcl2*); and (4) B cell (*Cd79a*, *Cd79b*, *Ly6d*, and *Ebf1*). Subsequently, we annotated the sub-populations of macrophage/monocyte. Based on the well-known gene markers, we classified the macrophage/monocyte population into six cell types [21]: (1) *Ly6c2*+ monocytes, as reflected by higher expression of *Ccr2*, *Ly6c2*, *Plac8*, and *Hp*; (2) MΦ-1, as reflected by higher expression of cardiac repair-related genes (*Il10*, *Trem2*, *Gpnmb*, *Timp2*, *Spp1*, and *Igf1*); (3) MΦ-2, as reflected by higher expression of early macrophage-specific genes (*Chil3* and *Clec4e*) and inflammatory response genes (*Il6*, *Il1b*, *Il18*, and *Nlrp3*); (4) MΦ-3, as reflected by higher expression of classic resident macrophage genes (*Lyve1*, *Gas6*, and *Cbr2*); (5) MΦ-4, as reflected by higher expression of cell cycle-related genes (*Stmn1*, *Cks1b*, *Birc5*, *Top2a*); and (6) MΦ-5, as reflected by higher expressions of antigen presentation-related genes (*H2-Ab1*, *H2-Aa*, *H2-Eb1*, *Cd74*). To understand the expression dynamics of MS across the transition of these macrophage subtypes, we initially performed pseudo-time analysis to uncover the differentiation trajectory of macrophages using the R package “monocle3” (version 1.3.4) [25]. We then projected the trajectory into a two-dimensional UMAP plot. Additionally, we identified differentially expressed genes (DEGs) in macrophage subtypes that varied across the pseudo-time (screening threshold: Moran’s I > 0.01 and Q-value < 0.05). Using the R package “ClusterGVis” (version 0.1.1) [26], the kinetic patterns of DEGs across the pseudo-time were further determined using the K-means approach. The GO and KEGG terms of distinct kinetic patterns were investigated using the R package “clusterProfiler” (version 4.8.1) [27]. Terms with *p*-values less than 0.05 were considered to be significant enrichments of DEGs.

### 2.6. Mendelian Randomization (MR) Analysis

By implementing MR analysis on MS genes, we could understand whether these genes could serve as AMI causative and therapeutic genes, which will largely enhance the treatment power of the MS model. We used the R package “TwoSampleMR” [28] to conduct a two-sample MR analysis to explore the causal association between *ITGB1* expression (exposure) and AMI risk (outcome). We retrieved the exposure data (ID: eqtl-a-ENSG00000150093) and outcome data (ID: ebi-a-GCST90038610) from the IEU Open GWAS data source (https://gwas.mrcieu.ac.uk/, accessed on 30 March 2024). Following the assumptions of MR [29], we initially retained single nucleotide polymorphisms (SNPs) that were significantly associated with *ITGB1* expression (*p* < 5 × 10^−6^) but not associated with AMI risk (*p* > 0.05); these were used as instrumental variables (IVs) in two-sample MR analysis. To avoid linkage disequilibrium (LD), we excluded the SNPs with LD-R2 greater than 0.01 within a cropping range of 10,000 Kb. Based on these SNPs, we subsequently harmonized the exposure and outcome data. Five approaches in two-sample MR, including MR Egger, weighted median, inverse variance weighted (IVW), simple mode, and weighted mode, were used to assess the causal association between the *ITGB1* expression and AMI risk. Leveraging Bayesian weighted MR (BWMR), we further made the causal inference as well as a validation of two-sample MR results. Wald ratio-based effects from two-sample MR and BWMR were shown as forest plots using the R package “forestploter” [30].

### 2.7. Candidate Drug Prediction and Molecular Docking

Assessing the protein–drug interaction helps us understand the druggability of a gene [31]. We predicted a panel of small-molecule drugs potentially interacting with ITGB1 using the Drug Signatures Database (DSigDB) (https://maayanlab.cloud/Enrichr/, accessed on 30 March 2024) [32], which is summarized in Supplementary Table S3. We focused on the top three significant drugs with higher combined scores: ML-9, ginsenoside Rh1, and WP1066. We selected ginsenoside Rh1, a natural anti-inflammatory agent, rather than specific inhibitors, as a candidate drug interacting with ITGB1. Next, we performed molecular docking, a well-established approach to computing protein–ligand interactions [33], to investigate the binding affinities and interactive modes between ITGB1 and ginsenoside Rh1. We downloaded the three-dimensional structure of ITGB1 (PDB format; accession number 4WK0) and the two-dimensional structure of ginsenoside Rh1 (SDF format) from the Protein Data Bank (https://www.rcsb.org/, accessed on 26 April 2024) and PubChem (https://pubchem.ncbi.nlm.nih.gov/, accessed on 26 April 2024), respectively. ChemBio3D software (version 14.0.0.17) was used to minimize the energy of ginsenoside Rh1 and then convert it to a three-dimensional structure (mol2 format). PyMOL software (version 2.4.0) was used to remove solvents, ligands, and hydrogens. Using the AutoDock Vina software (version 1.1.2), we then converted the mol2 format of ginsenoside Rh1 into PDBQT format for further docking. Additionally, the AutoDock Vina software was employed to execute polar hydrogenation and docking site settings, and it subsequently converted the PDB format of ITGB1 into the PDBQT format for docking ginsenoside Rh1. The parameters of the ITGB1 docking site, including X-Y-Z coordinates and grid size, were set to encompass ginsenoside Rh1. Subsequently, AutoDock Vina software was used to dock ITGB1 and ginsenoside Rh1 20 times. The optimal docking performed with the lowest binding affinity and Root Mean Squared Error (RMSE) lower than 2 Å was retained and visualized [34] to demonstrate ITGB1’s docking efficiency to ginsenoside Rh1.

## 3. Results

### 3.1. An Integrative ML-Based Framework Develops a Roust Migrasome-Related Signature (MS) to Predict AMI

**Figure 2:**
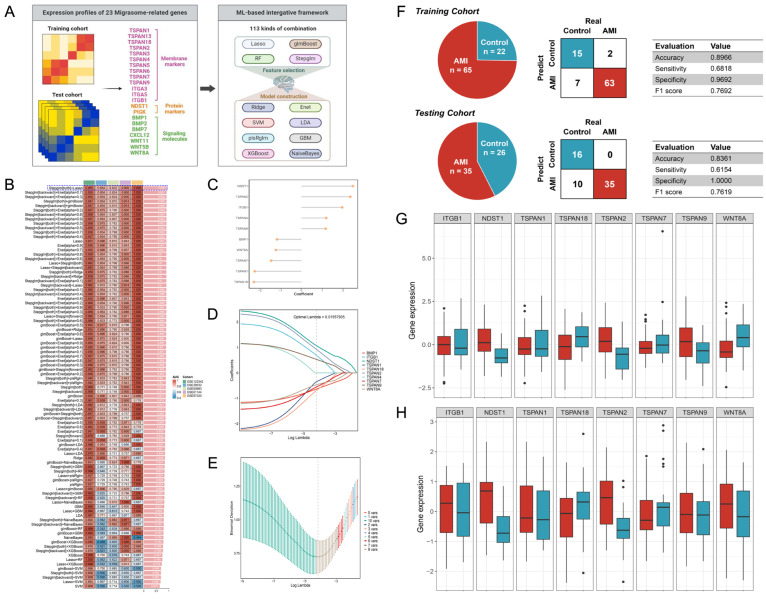
Development of an MS for predicting AMI. (**A**) ML-based integrative framework for establishing the MS. (**B**) AUC scores of 113 ML combinations within the training and testing cohorts. The best-performing ML combination (first-ranked) is highlighted in the blue box. (**C**) Coefficient profiles of ten genes were obtained in the Stepglm algorithm. (**D**) Lasso further selected nine genes with non-zero coefficients under the optimal lambda. (**E**) The optimal lambda was obtained when the minimum deviation was reached. (**F**) Overview of the control and AMI cases within the training and testing cohorts (left). MS-derived confusion matrix and predictive indicators (right). (**G**) Expressions of nine genes between the control and AMI in the training cohort. (**H**) Expressions of nine genes between the control and AMI in the testing cohort.

We initially collected a list of 35 migrasome-related genes (Supplementary Table S1). To ensure comparability, we subsequently retained 23 migrasome-related genes that overlapped within the five independent cohorts (Figure 2A): *BMP1*, *BMP2*, *BMP7*, *CXCL12*, *ITGA3*, *ITGA5*, *ITGB1*, *NDST1*, *PDGFD*, *PIGK*, *TSPAN1*, *TSPAN13*, *TSPAN18*, *TSPAN2*, *TSPAN3*, *TSPAN4*, *TSPAN5*, *TSPAN6*, *TSPAN7*, *TSPAN9*, *WNT11*, *WNT5B*, and *WNT8A*. Among these cohorts, GSE123342, with the largest number of samples, was selected as our training cohort for developing an MS, while GSE29532, GSE60993, GSE61144, and GSE97320 were the independent testing cohorts for signature validation. Next, expression profiling of 23 migrasome-related genes was subjected to an integrative ML-based computational framework to establish an MS. We employed 12 ML algorithms and fitted 113 algorithmic combinations. As shown in Figure 2B, the optimal ML combination, consisting of Stepglm (direction = both) and Lasso, performed with the highest average AUC score (0.908) across the five cohorts. We called this the optimal combination MS. The progress of developing the MS is depicted in Figure 2C–E. Using Stepglm (direction = both) as a selection, we screened out ten feature genes from 23 migrasome-related genes (Figure 2C). Subsequently, nine genes with nonzero Lasso coefficients—*BMP1*, *ITGB1*, *NDST1*, *TSPAN1*, *TSPAN18*, *TSPAN2*, *TSPAN4*, *TSPAN7*, *TSPAN9*, and *WNT8A*—were identified (Figure 2D,E). Additionally, the training and validation loss curve was leveraged to assess the performance of MS in predicting AMI, as depicted in Supplementary Figure S1. This loss curve demonstrates that the learning process of MS was converging for about 40 epochs, suggesting a good fit with a minimal gap between the final training and validation losses. Employing the nine genes-based MS, we subsequently predicted the probability of each case within the training and testing cohorts. The case distribution and confusion matrices of the training and testing cohorts are outlined in Figure 2F. Notably, we also calculated four well-known evaluation metrics, including accuracy, sensitivity, specificity, and F1 score, to verify the robust predictive performance of MS. We then investigated the expression patterns of nine genes among the control and AMI cases (Figure 2G,H). The expressions of *ITGB1*, *NDST1*, *TSPAN1*, *TSPAN2*, *TSPAN4*, and *TSPAN9* were upregulated in AMI, whereas *TSPAN18* and *TSPAN7* were downregulated.

### 3.2. Comparison of Predictive Performance between the MS and Published Signatures

**Figure 3:**
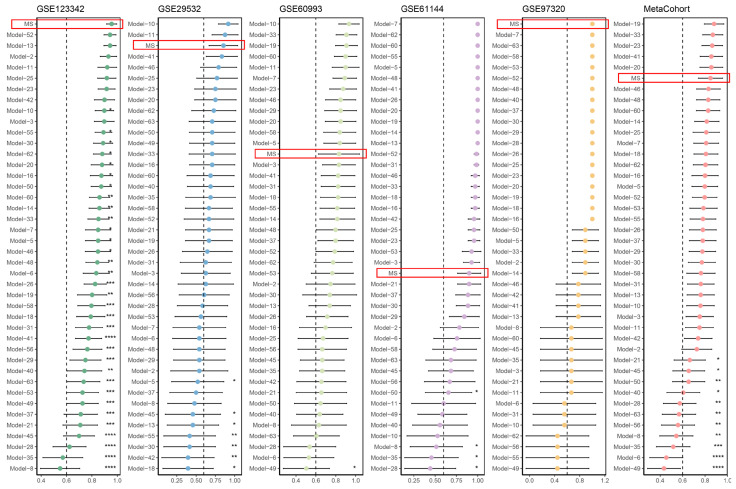
Comparison of predictive performance between the MS and published signatures. The AUC scores of the MS model within five cohorts and the meta-cohort are highlighted in red boxes. An unpaired student’s test was performed; * *p* < 0.05, ** *p* < 0.01, *** *p* < 0.001, **** *p* < 0.0001.

Massive predictive signatures have been extensively reported with advancements in omics technologies and computational approaches. To enable equitable comparability between our MS and other signatures, we systemically conducted a literature review and ultimately incorporated 63 mRNA signatures of AMI prediction (Supplementary Table S2). Notably, these signatures were associated with diverse biological functions, such as immune infiltration, cell death, and the extracellular matrix. We filtered out published signatures with more than 30% mismatched genes within the five cohorts. We retained a final set of 42 signatures for comparison. As depicted in Figure 3, we noted that MS demonstrated better AUC performance than most of the other published signatures. The AUC scores of MS ranked first in the GSE123342 and GSE97320 cohorts, while they were third in the GSE29532 cohort. MS performed relatively weakly in the GSE60933 and GSE61144 cohorts. Nonetheless, our MS retained robust performance in the meta-cohort that merges all cohorts, suggesting a better generalization ability than other signatures.

### 3.3. Macrophage-Specific Expression Patterns of MS in AMI at Single-Cell Resolution

**Figure 4:**
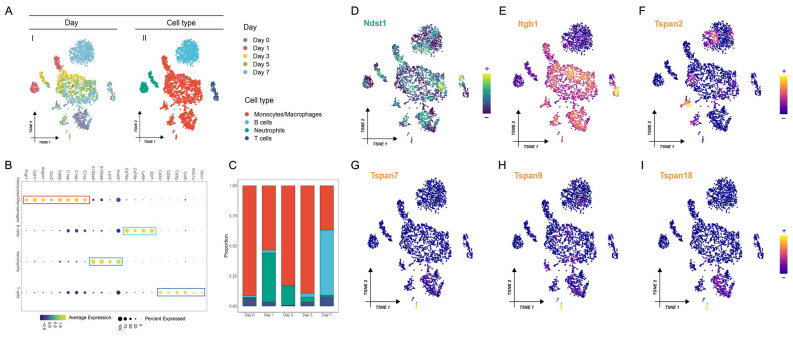
Expression patterns of MS at single-cell resolution in AMI progression. (**A**) t-SNE visualization of immune cells from control and infracted murine hearts, with annotated time points and cell type. (**B**) Dot plot of marker genes for five cell types. The scale colors represent the average expression levels of marker genes. The dot size represents the percentage of cells expressed within the cell types. The box with different colors stands for a panel of specific gene markers for four cell populations. (**C**) The numbers of each cell type in control (Day 0) and different time points after AMI (Days 1, 3, 5, and 7). Expression distribution of *Ndst1* (**D**), *Itgb1* (**E**), *Tspan2* (**F**), *Tspan7* (**G**), *Tspan9* (**H**), and *Tspan18* (**I**) in five cell types. *Itgb1* was more highly expressed in macrophages than in the other four cell types.

To delve deeper into the biological mechanism of MS, we implemented scRNA-seq analysis on cardiac *Cd45*+ cells isolated from murine models with AMI (Day 0, Day 1, Day 3, Day 5, Day 7). After performing quality control and filtering, we gained 17, 384 high-quality cells with higher *Cd45* expression. Using well-established cell markers as annotation, we successfully identified four cell populations: macrophage/monocyte, T/NK cell, neutrophil, and B cell. Single-cell profiling of cardiac *Cd45*+ cells, including different time points and cell types, is depicted in Figure 4A. Detailed information on these cell markers’ expression in the four cell populations is summarized in Supplementary Figure S3. Notably, the macrophage/monocyte population was the most abundant, accounting for more than half of the cells. The overall expression distribution of cell markers among the cell populations is shown in Figure 4B. To investigate the dynamic alterations in cells, we calculated the proportion of each cell population at different time points. As shown in Figure 4C, the macrophage/monocyte population showed a dramatic decrease on day one after AMI, whereas there was an incremental expansion from day three to day five post-AMI. Subsequently, we discriminated the cell population with relatively higher expressions of MS. We detected the expression patterns of six MS genes among these cell populations, including *Ndst1* (Figure 4D), *Itgb1* (Figure 4E), *Tspan2* (Figure 4F), *Tspan7* (Figure 4G), *Tspan9* (Figure 4H), and *Tspan18* (Figure 4I). Intriguingly, we noted that *Itgb1* was significantly upregulated in the macrophage/monocyte population (Figure 4E), suggesting the potential of *Itgb1* in regulating macrophage/monocyte activities during AMI progression.

### 3.4. Identification of Dynamic MS Genes during the Transition of Macrophages

**Figure 5:**
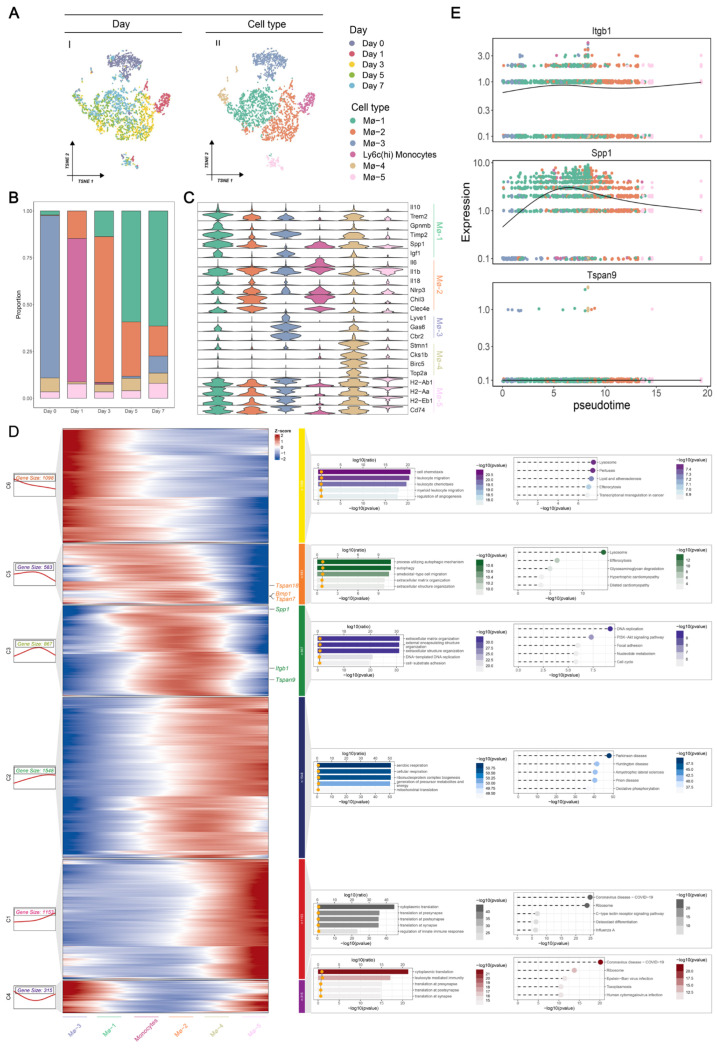
Pseudo-time analysis reveals the dynamics of MS during macrophage transition. (**A**) t-SNE visualization of macrophages from control and infracted murine hearts, with annotated time points and macrophage type. (**B**) The numbers of each macrophage type in control (Day 0) and different time points after AMI (Day 1, Day 3, Day 5, Day 7). (**C**) Violin plot of marker genes of five macrophage types. (**D**) Heatmap of dynamic genes during the macrophage transition (left). Significant GO and KEGG enrichment terms for dynamic genes (right). (**E**) Trajectory plots showing the dynamics of *Itgb1*, *Ssp1*, and *Tspan9* in macrophage differentiation ordered by pseudo-time.

We previously demonstrated the specific upregulation of *Itgb1* in the macrophage/monocyte population. Afterward, we concentrated on the dynamic characteristics of *Itgb1* and its interplay with macrophages/monocytes. Given the heterogeneous population and diverse biological functions, we implemented second-level clustering on the macrophage/monocyte population. In total, we identified six cell populations based on the well-known cell markers, including *Ly6c2*+ monocytes, MΦ-1, MΦ-2, MΦ-3, MΦ-4, and MΦ-5 (Figure 5A). The proportion of each cell population at different time points is shown in Figure 5B. Notably, *Ly6c2*+ monocytes expand on day one after AMI, whereas they significantly diminish on day 3. Additionally, MΦ-2 mainly appeared on day one and accumulated on day 3, indicating its pro-inflammatory role in the early stage. In contrast, we noted that MΦ-1 progressively increased from day 3 to day 7, suggesting its anti-inflammatory function in the late stage. We initially identified *Ly6c2*+ monocytes using a combination of well-established markers (*Ly6c2*+, *Ccr2*+, and *Cx3cr1*−), which were absent from the macrophage population. Next, we annotated five subpopulations of macrophages. The expression distribution of cell markers in these macrophage subpopulations is visualized in Figure 5C. Detailed information on these cell markers’ expressions among the macrophage subpopulation is summarized in Supplementary Figure S4. We observed that MΦ-1 was reflected by higher expression of cardiac repair-related genes, such as *Il10*, *Trem2*, *Gpnmb*, *Timp2*, *Spp1*, and *Igf1*, while MΦ-2 expressed high levels of early macrophage-specific genes (*Chil3* and *Clec4e*) and pro-inflammatory response genes (*Il6*, *Il1b*, *Il18*, and *Nlrp3*). Together with the dynamic alteration in proportion, we showed the anti-inflammatory subset (MΦ-1) and pro-inflammatory subset (MΦ-2) of macrophages in AMI progression. MΦ-3 exhibited higher expression of classical resident macrophage genes (*Lyve1*, *Gas6*, and *Cbr2*) manifestly diminished after AMI occurrence but reappeared on day 7. MΦ-4 was characterized by high expression of cell cycle genes (*Stmn1*, *Cks1b*, *Birc5*, and *Top2a*). MΦ-5 showed the ability to participate in antigen presentation and processing, as reflected by higher *H2-Ab1*, *H2-Aa*, *H2-Eb1*, and *Cd74* expression.

To better understand the cell dynamics and transitions, we performed pseudo-time analysis to infer the differentiation trajectory of macrophages. We also investigated the kinetic patterns of genes that were differentially expressed across the pseudo-time, displayed in Figure 5D (left panel). We identified six different kinetic patterns (denoted by C1–C6) and each pattern was determined using the GO and KEGG enrichment analyses (Figure 5D, right panel). We found that *Tspan18*, *Bmp1*, and *Tspan7* were allocated to C5, which exhibited decreasing expression along the pseudo-time. Enrichment analysis showed that C5 was significantly associated with lysosome and autophagy functions. More importantly, *Spp1*, *Itgb1*, and *Tspan9* were clustered in C3, demonstrating a bimodal expression pattern across pseudo-time (Figure 5E). Enrichment analysis further showed that C3 was involved in biological processes related to extracellular matrix organization and DNA replication. Specifically, we depicted the dynamics of *Spp1*, *Itgb1*, and *Tspan9* across the pseudo-time. As shown in Figure 4E, *Itgb1* and *Spp1* represent the bimodal expression pattern of C3, with MΦ-1 mainly expressed and MΦ-2 slightly decreased. Tspan9 showed a lower expression level in macrophages, apart from the conventional C3 pattern. Taken together, we observed that *Itgb1* was dynamically expressed through a switch from the pro-inflammatory phase of MΦ-2 to the anti-inflammatory phase of MΦ-1.

### 3.5. Causal Effect and Druggability of ITGB1 on AMI

**Figure 6:**
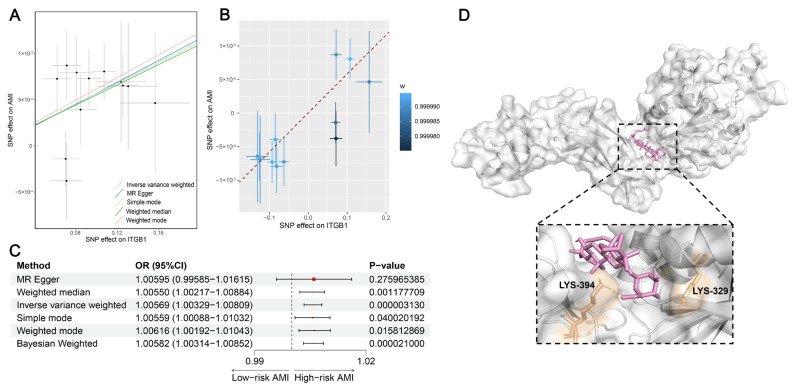
The causal association and potential druggability of *ITGB1* and AMI risk. (**A**) Two-sample Mendelian randomization analysis of the positive association between the SNP effect on *ITGB1* (x-axis) and AMI (y-axis). (**B**) Bayesian weighted Mendelian randomization analysis of the positive association between the SNP effect on *ITGB1* (x-axis) and AMI (y-axis). (**C**) Forest plot displaying the OR effects (95% confidence intervals) and *p*-values of *ITGB1* expression on AMI risk. (**D**) Molecular docking visualization of ginsenoside Rh1 and ITGB1.

Given that *ITGB1* was upregulated in AMI and dynamically altered along the macrophage transition from pro-inflammatory to anti-inflammatory, we sought to explore the causal effect and druggability of *ITGB1* on AMI. We initially performed a two-sample MR analysis using the two GWAS datasets (see Methods). Following the MR assumption, we selected 12 SNPs as strong IVs to assess the causal relationship between *ITGB1* expression and AMI risk. As depicted in Figure 6A, we related the positive effect size of the SNP-*ITGB1* relationship to the SNP–AMI relationship. We further verified their positive relationship using the BWMR approach (Figure 6B). Using the IVW method, with the highest statistical power, we observed that a genetic predisposition toward higher expression of *ITGB1* significantly elevated the AMI risk (OR > 1, *p* < 0.05; Figure 6C). Moreover, we noted the same direction of estimated effect in the other four approaches and BWMR. To guarantee the reliability of MR, we conducted sensitivity analyses. We did not observe heterogeneity and horizontal pleiotropy (Supplementary Table S4).

We used MR analysis to demonstrate that *ITGB1* expression increased the AMI risk. Given the role of *ITGB1* as a potential AMI-causative gene, discovering a drug that strongly binds to ITGB1 may contribute to alleviating the AMI condition. We used the DSigDB database to predict ginsenoside as a candidate drug interacting with ITGB1. To measure the affinity of the ITGB1–ginsenoside Rh1 interaction and from this to understand the druggability of ITGB1, we implemented the AutoVina software (version 1.1.2) to execute the molecular docking. As shown in Figure 6D, we visualized the docking model that the binding pocket of ITGB1 could successfully encompass the structure of ginsenoside, of which ginsenoside interacts with the amino acid bases LYS-329 and LYS-394 of ITGB1. Overall, we demonstrated the great potential of *ITGB1* as an AMI therapeutic target.

## 4. Discussion

Despite the advancements in diagnosis and treatment, AMI remains high in morbidity and mortality worldwide [1]. The risk of mortality appears to be highest within the first hours of the onset of AMI; therefore, a prompt and accurate diagnosis of AMI is essential to improving this situation. Nevertheless, the clinically available AMI biomarkers, characterized by cTn, are still far from ideal for AMI diagnosis [5]. Migrasome, a novel multi-functional EV, has been proven to play essential roles in diverse biological processes, including cell communication, homeostasis maintenance, and embryonic development [35]. Although some studies have also underscored the underlying role of migrasomes in disease occurrence, progression, and diagnosis [35], their clinical applications have yet to be fully explored. Owing to the great advancement in bioinformatics, increasing evidence points to the omics-based screening of disease biomarkers. Therefore, we collected microarrays of AMI patients to explore the diagnostic significance of migrasomes.

To the best of our knowledge, this is the first attempt to develop a predictive migrasome-related signature for AMI patients. Five independent microarrays from 100 AMI patients were adopted for the construction of an MS. Though ML approaches contribute to the development of multigene expression-based signatures for predicting disease occurrence and progression, some apparent shortcomings such as inappropriate selection of modeling samples and personal preference of ML algorithms still needed be remedied in further investigations [36]. Notably, we meticulously curated peripherical blood-derived microarrays as single-source expression profiling, which greatly guarantees the specificity and generalizability of our analyses. Gene signatures generated from single-sample source datasets ensure the robustness of analytical results. At the same time, those derived from “mixed” samples that ignore biological significance usually underfit in modeling, such as an incorrect integrative analysis using datasets collected from myocardial tissues, PB, and peripheral blood mononuclear cells [37]. Further, in contrast to most of the previous AMI-predictive signatures based on a specific biological process, we systemically collected a panel of reported migrasome-related genes as feature inputs in signature development, including membrane markers, protein markers, and signaling molecules. As found in our modeling investigation, by introducing more pivotal features that strongly characterize the migrasome, we were able to construct a robust predictive signature. In the other aspect, most of the underfitting gene signatures were directly constructed with a single ML classifier lacking parameter tuning, which is mainly attributed to the bias of approach selection [36]. To remedy this underfit trouble caused by personal preference for an algorithm, we collected 12 prevalent binary classification ML algorithms and packed them into 113 combinations to enhance modeling complexity. Leveraging this integrative ML computational framework established on 10-fold cross-validation, we could reduce the dimensionality of migrasome-related genes, thereby establishing a more feasible and simplified signature that fits well. Additionally, we previously demonstrated the robustness and simplicity of this framework for identifying disease biomarkers [23]. Ultimately, the optimal combination (namely MS) was generated via integrating Stepglm and Lasso algorithms, which showed the highest average AUC performance (0.908) in the five independent AMI cohorts. More specifically, the MS was a panel of nine migrasome-related genes that were most potent in predicting AMI: *BMP1*, *ITGB1*, *NDST1*, *TSPAN1*, *TSPAN18*, *TSPAN2*, *TSPAN4*, *TSPAN7*, *TSPAN9*, and *WNT8A*.

Overfitting is a troublesome issue encountered when constructing predictive signatures, with several models demonstrating excellent performances in their training cohorts but extremely poor performance in external cohorts [38]. Using an algorithmic combination of Stepglm and Lasso on migrasome features, we finally obtained an optimal nine gene-based signature termed MS, which showed the highest average AUC performance within the training cohort and the other four independent testing cohorts. Due to the effectiveness of feature selection power, the integration of Stepglm and Lasso could minimize redundant information and identify the most important features for establishing a well-fitted MS. To subsequently investigate the generalizability and extensibility of MS, we retrieved other published signatures for comparison. We enrolled a total of 63 published signatures for predicting AMI, composed of various functional gene combinations. Compared with these signatures, our MS was shown to precisely detect AMI with the leading AUC performances (larger than 0.8 in all cohorts), presenting better generalization capability than other signatures. Of these signatures, some present poor generalizability, and very few have been rigorously validated (Table 2). For example, signature 10 demonstrated better AUC scores in the GSE123342, GSE29532, and GSE60993 cohorts but was unsatisfactory in others. Also of note is that the Protein–Protein Interaction (PPI) method accounts for over half of the final signatures (22/42) compared to MS. However, most displayed poor generalizability across these cohorts; this can be attributed to features with high correlations. Though PPI helps us to identify hub genes from a functional protein-associated network of great biological significance [39], it may cause overfitting problems in signature constructions brought by the mRNA expression levels of these highly correlated proteins. Conversely, our MS model presents better generalizability, which primarily benefits from our meticulously designed ML framework. Notably, the Lasso model was the final and optimal one for building MS. To tackle underfitting and overfitting troubles, it introduces a penalty term (L1 regularization) to regulate complexity [40]. L1 regularization contributes to producing sparse solutions, which could reduce the model complexity to prevent overfitting and eliminate some non-prominent features by shrinking their coefficients toward zero. Additionally, the hyper-parameter lambda that controls L1 regularization was optimized using the 10-fold cross-validation, thereby establishing a well-fitted signature that is well-balanced overfitting and underfitting. This may explain why our MS fitted by Lasso showed better generalizability than other published signatures. Altogether, we successfully established an MS model with the potential to predict AMI occurrence.

To understand the latent biological mechanisms of MS, we implemented scRNA-seq analysis on cardiac *Cd45*+ cells isolated from murine models of AMI. We annotated five cell populations using well-established marker genes, including macrophage/monocyte, T/NK cell, neutrophil, and B cell. We noted that the macrophage and monocyte were the most abundant cell populations, accounting for more than 50% of all cells. Additionally, the macrophage/monocyte population decreased on the first day after AMI, but increasingly recovered from day 3 to day 7, which agrees with previous evidence [41]. Subsequently, we investigated the expression patterns of MS across the five cell populations. Among MS composed of nine genes, *ITGB1* was significantly expressed in the macrophages/monocytes. This data suggests the specific upregulation of *ITGB1* in cardiac macrophages and monocytes in AMI. Afterward, we detected six sub-populations of macrophages/monocytes: *Ly6c2*+ monocytes, MΦ-1, MΦ-2, MΦ-3, MΦ-4, and MΦ-5. Anti-inflammatory MΦ-1 and pro-inflammatory MΦ-2 represent divergent functional populations. The dynamics of MΦ-1 and MΦ-2 also demonstrate the opposite pattern, in which MΦ-2 sharply expanded at the onset of AMI but then decreased. While MΦ-1 represents an anti-inflammatory role, it dramatically increased from day 3 to day 5 after AMI. In addition, single-cell trajectories based on pseudo-time were used to uncover the association between the gene expression changes and cell transitions. In total, we uncovered six kinetic gene expression patterns among the macrophages. Enrichment analysis further showed that these patterns stand for diverse biological process transitions. Of note, we found that *ITGB1* kept changing during the differentiation of macrophages, especially MΦ-1 and MΦ-2 populations. These data indicated the regulatory role of *ITGB1* in the macrophage transformation between the pro-inflammatory and anti-inflammatory phases. Macrophages orchestrate the resolution of inflammation following AMI by clearing neutrophils and myocardial debris [42]. ITGB1 is a known phagocytic receptor widely expressed on the surface of macrophages and involved in regulating inflammatory responses [43]. This evidence was consistent with our study. Upon AMI, damaged cardiomyocytes release extracellular matrix (ECM) components, such as fibronectin and collagen. Then, ITGB1 acts with the ITGB5 subunit to form a heterodimeric receptor, which transduces fibronectin-mediated signals through the Arg-Gly-Asp (RGD) motif, facilitating macrophage adhesion and migration [44]. Activated macrophages, via ITGB1-mediated FAK and PI3K/AKT signaling pathways, release inflammatory cytokines, including tumor necrosis factor-alpha (*TNF-α*), interleukin-1 beta (*IL-1β*), and interleukin-6 (*IL-6*), further amplifying the inflammatory response and attracting additional immune cells to the infarcted area [45]. After AMI, the prolonged survival of macrophages in the damaged area enhances their efficiency in clearing necrotic tissue and promoting tissue repair. Additionally, *ITGB1* facilitates myocardial regeneration and scar tissue formation by regulating macrophages and other immune cells to secrete growth factors and cytokines, thereby promoting angiogenesis and fibroblast activation [46]. Overall, this evidence broadly supports our findings about the regulatory role of *ITGB1* in transforming the pro-inflammatory to anti-inflammatory phases of macrophages. Nevertheless, the molecular mechanism underlying *ITGB1* across macrophage transition is still worth validating, especially in terms of how it activates the migrasome.

Subsequently, we explored the causality between *ITGB1* expression and AMI risk. Using two-sample MR as a primary analysis and BWMR as validation, the increase in *ITGB1* expression was found to elevate the risk of AMI. Existing research has found that *ITGB1* is involved in cardiac remodeling post-MI, which is essential for cardiac adaptation to myocardial ischemia and the prevention of AMI [47]. Therefore, the expression level of *ITGB1* serves as a biomarker for assessing the severity and prognosis of MI while modulating its expression can significantly influence cardiac function repair after AMI. As a vital member of the transmembrane glycoprotein receptor family involved in bidirectional intracellular and extracellular signal transduction, *ITGB1*’s complex signaling network makes it a vital therapeutic target. Next, we predicted ginsenoside as a candidate drug interacting with ITGB1. Based on the molecular docking, we further demonstrated the close and stable bindings between ITGB1 and ginsenoside via the formation of hydrogen bonds, suggesting the druggability of ITGB1. Recent research has revealed that salvianolic acid A improves survival cell rates in an MI model by inhibiting ITGB1 and its associated signaling pathways [48]. Besides, volociximab, a monoclonal antibody targeting ITGB1, has demonstrated potential in anti-cardiovascular disease. Therapeutic strategies targeting ITGB1 have demonstrated potential in basic research and paved the way for novel approaches in clinical treatment. Developing ITGB1-based drugs may provide more precise and effective therapeutic interventions, improving the prognosis of patients with MI and other cardiovascular diseases. However, further clinical research is needed to elucidate the specific mechanisms of ITGB1 in different pathological states and optimize its regulatory strategies to ensure the effectiveness of treatment.

Notably, our work differed from previous investigations in three aspects: (1) We curated five independent AMI datasets derived from PB, as a single-source sample analysis, to guarantee specific signature establishment using migrasome features. Developing a signature from a single-source sample such as PB worked well in our investigation, and provides a methodology reference for other researchers. (2) Unlike current signature studies based on personal preferences and biases, we systemically collected 12 prevalent bi-classification ML approaches and packed them into 113 algorithmic combinations, which generated an optimal one with the best predictive performance, termed MS. Leveraging this integrative ML framework, we could quickly and accurately establish a predictive signature from a selection of massive features of unknown significance, and offers a valuable reference tool for the exploration of other signatures. (3) From a single-cell resolution level, we expounded the latent biological mechanisms underlying MS, which is from a macrophage transition between pro-inflammatory and anti-inflammatory. We also suggested a positive causal relationship between MS expression and AMI risk. Investigating the latent biological mechanism of signature from the single-cell RNA-seq datasets, including expression pattern and pseudo-time analysis, provides valuable insights for understanding their functional roles. Additionally, we proposed an analytical workflow, combining MR and molecular docking, to inquire into the genetic casualty and therapeutic value of the signature. From this workflow, we could understand whether a signature could serve as a therapeutic target and may guide the optimization of clinical decisions.

Although the clinical and biological significances of MS in AMI have been systemically and rigorously illuminated, several limitations remain to enlighten further work. First, the collected samples were retrospective, and further validation in prospective multicenter cohorts with larger samples is warranted. Compared with our traditional ML models, more eligible deep learning algorithms should be enrolled for signature establishment in the future. Second, the roles of nine genes in AMI are still unclear, and more functional experiments, such as gene knockout and pharmaceutical effects, are required to validate our in-silicon data. Third, it should be noted that molecular or clinical traits such as genomic mutations, pathological type, and gender were inadequate in our enrolled cohorts, which may conceal underlying correlations between MS and specific traits. Thus, combining our MS with several traits may enhance the model’s robustness and prediction accuracy. This is worth studying further.

## 5. Conclusions

We established an MS to predict AMI, demonstrating superior predictive performance and generalization capability within the five independent cohorts. MS was an algorithmic integration of Stepglm and Lasso fitted to the expressions of nine migrasome-related genes: *BMP1*, *ITGB1*, *NDST1*, *TSPAN1*, *TSPAN18*, *TSPAN2*, *TSPAN4*, *TSPAN7*, *TSPAN9*, and *WNT8A*. As a simplified and powerful nine-gene-based signature with excellent performance, clinical laboratories may use this tool to diagnose AMI in future applications. Leveraging scRNA-seq of cardiac *Cd45*+ cells from inducible MI mouse models, we found that *ITGB1* was significantly expressed in cardiac macrophages. Additionally, the pseudo-time analysis uncovered the regulatory role of *ITGB1* in the macrophage transition from the anti-inflammatory to the pro-inflammatory phase, which proposed a candidate regulatory gene in cardiac macrophage functions for further investigation. We found a positive causality between *ITGB1* expression and AMI risk by performing MR analysis. Finally, we predicted ginsenoside as a therapeutic drug targeting ITGB1, indicating the druggable value of ITGB1 in curing AMI. Given that *ITGB1* is a therapeutic gene of AMI, repressing *ITGB1* overexpression via ginsenoside in AMI may represent a promising therapeutic approach. Thus, our MS model has significant applications in AMI occurrence, progression, and therapeutic response, and will inform future diagnostic and therapeutic strategies.

## Figures and Tables

**Table 1:** Detailed information on the AMI datasets enrolled in this study.

GSE Accession	Specimen Source	Platform	Control Cases	AMI Cases	Usage
GSE123342	PB	GPL17586	22	65	Training cohort
GSE29532	PB	GPL5175	6	8	Testing cohort
GSE60993	PB	GPL6884	7	17	Testing cohort
GSE61144	PB	GPL6106	10	7	Testing cohort
GSE97320	PB	GPL570	3	3	Testing cohort
GSE163465	*Cd45*+ cells	GPL24247	1	3	scRNA-seq cohort

**Table 2:** Comparisons between the MS and other published signatures.

Model No.	AUC Performance in						ML Algorithms/ Bioinformatics Approaches
	GSE123342	GSE29532	GSE60993	GSE61144	GSE97320	Meta- Cohort	
MS	0.955	0.854	0.832	0.900	1.000	0.848	Stepglm, LASSO
2	0.930	0.542	0.748	0.786	0.889	0.726	Univariate Regression, LASSO
3	0.897	0.625	0.832	0.929	0.667	0.753	RF, Artificial Neural Network (ANN)
5	0.849	0.521	0.840	1.000	0.889	0.798	Monte Carlo feature selection, incremental feature selection, SVM
6	0.834	0.542	0.529	0.757	0.556	0.455	Logistic Regression
7	0.849	0.542	0.891	1.000	1.000	0.810	Protein-protein interaction (PPI)
8	0.550	0.479	0.630	0.514	0.667	0.544	LASSO
10	0.898	0.917	0.933	0.529	0.556	0.759	PPI
11	0.921	0.875	0.899	0.600	0.667	0.751	PPI
13	0.943	0.458	0.739	1.000	0.778	0.762	PPI
14	0.859	0.625	0.815	1.000	0.889	0.815	PPI
16	0.875	0.708	0.697	0.971	1.000	0.800	PPI
18	0.791	0.396	0.824	0.971	1.000	0.808	Weighted Gene Co-expression Network Analysis (WGCNA)
19	0.801	0.667	0.908	1.000	1.000	0.880	SVM
20	0.880	0.750	0.849	1.000	1.000	0.856	PPI
21	0.708	0.667	0.655	0.900	0.667	0.664	NetworkAnalyst, PPI
23	0.916	0.750	0.874	0.957	1.000	0.864	WGCNA, PPI
25	0.917	0.771	0.672	0.957	1.000	0.811	PPI
26	0.825	0.646	0.714	1.000	1.000	0.780	Differentially-Expressed Genes (DEGs) analysis
28	0.624	0.583	0.538	0.443	1.000	0.575	PPI
29	0.749	0.542	0.849	0.843	1.000	0.776	PPI, Transcription Regulatory Network (TRN)
30	0.888	0.417	0.739	0.886	1.000	0.771	ANN
31	0.778	0.625	0.824	0.986	0.556	0.763	RF, SVM
33	0.852	0.708	0.908	0.971	0.889	0.869	PPI
35	0.571	0.688	0.664	0.457	0.667	0.516	LASSO, RF
37	0.712	0.500	0.798	0.886	1.000	0.779	DEGs, WGCNA
40	0.741	0.688	0.639	0.557	1.000	0.607	PPI
41	0.773	0.833	0.824	1.000	0.778	0.856	PPI
42	0.899	0.396	0.655	0.957	0.778	0.738	PPI, TRN
45	0.699	0.458	0.664	0.686	0.667	0.655	PPI
46	0.848	0.792	0.849	0.971	0.778	0.831	PPI
48	0.843	0.542	0.798	1.000	1.000	0.829	DEGs analysis
49	0.726	0.708	0.504	0.586	0.444	0.434	PPI, Fuzzy C-Means Clustering (FCM)
50	0.873	0.708	0.647	0.657	0.889	0.654	PPI
52	0.943	0.667	0.790	0.986	1.000	0.797	PPI
53	0.726	0.563	0.765	0.929	1.000	0.786	WGCNA
55	0.891	0.417	0.815	1.000	0.444	0.781	WGCNA, PPI
56	0.764	0.604	0.664	0.671	0.444	0.559	LASSO, Support Vector Machine Recursive Feature Elimination (SVM-RFE)
58	0.796	0.667	0.840	0.729	1.000	0.764	LASSO, SVM-RFE
60	0.860	0.688	0.899	1.000	0.667	0.827	LASSO, SVM-REF, RF
62	0.882	0.729	0.773	1.000	0.444	0.804	WGCNA
63	0.735	0.708	0.605	0.686	1.000	0.568	Traditional AMI markers

## Data Availability

The GEO databases (http://www.ncbi.nlm.nih.gov/geo, accessed on 10 January 2024) contain the datasets (GSE29532, GSE60993, GSE61144, GSE97320, and GSE163465) used to support this study.

## Supplementary Materials

The following supporting information can be downloaded at: https://www.mdpi.com/article/10.3390/biomedicines12071626/s1, Figure S1: The training and validation loss curve of MS. Figure S2: Pre-processing of scRNA-seq data; Figure S3: Annotation of four cell populations; Figure S4: Annotation of macrophage/monocyte population; Table S1: A total of 32 reported migrasome-related genes; Table S2: A total of 63 published signatures retrieved from the literature; Table S3: Predicted drugs interacting with ITGB1; Table S4: Sensitivity analyses; File S1: Literature review of published migrasome-related genes [8,9,49,50,51,52,53,54,55,56,57,58,59,60,61,62,63,64,65,66,67,68,69,70,71,72,73] and AMI signatures; File S2: Supplementary Methods.
