# Supplementary material for: Unveiling the Potential of Migrasomes: A Machine-Learning-Driven Signature for Diagnosing Acute Myocardial Infarction
Source: Biomedicines. 2024 Jul 22;12(7):1626. doi: 10.3390/biomedicines12071626 (PMC11274667; doi:10.3390/biomedicines12071626)
Supplement: Supplementary file 1 [file biomedicines-12-01626-s001.zip › biomedicines-3087802-supplementary/Supplementary File S1.pdf]

Literature review of reported migrasome genes

Current research has confirmed that migrasomes are composed of TSPANs, cholesterol, integrins, and other unidentified molecules, with TSPANs and integrins being highly enriched as membrane markers within the migrasomes[1].Among TSPANs, the overexpression of TSPAN1, 2, 3, 4, 5, 6, 7, 9, 13, 18, 25, 26, 27, and 28 promotes migrasome formation, with TSPAN1, 2, 4, 6, 7, 9, 18, 27, and 28 exhibiting particularly strong effects[2].TSPAN4 is one of the most effective migrasome-inducing tetraspanins and acts as a distinct migrasome marker, exhibiting a high capacity to enhance migrasome formation; deletion of Tspan4 in MG803 cells markedly decreases migrasome formation[3].TSPAN1 has been identified as a cancer-associated cell migration protein whose upregulation enhances prostate cancer cell migration, potentially by modulating migrasome formation to influence tumor cell motility and metastasis, making it a promising target for cancer research[4].TSPAN2 is expressed in vascular smooth muscle cells, and its expression level decreases following atherosclerosis or blood flow obstruction, playing a critical role in the pathogenesis of occlusive vascular diseases[5].In acute myeloid leukemia research, TSPAN3 is considered a critical signaling molecule, and its deletion impairs leukemia cell migration towards stromal cell-derived factor-1[6].TSPAN5 is abundantly expressed in vascular endothelial cells and is pivotal in modulating angiogenesis, likely by influencing endothelial cell proliferation, migration, and differentiation to facilitate neovascularization[7].Recent studies have found that overexpression of TSPAN6 in glioblastoma cells enhances the migratory capacity of vascular endothelial cells[8].Knockout of ITGB1 and TSPAN7 genes in zebrafish reveals a significant reduction in migrasome formation and results in organ morphology defects and left-right asymmetry errors during embryonic development[9].In bone marrow-derived macrophages from Tspan9$^{-/-}$ mice, the formation of migrasome is significantly reduced, further confirming the critical role of TSPAN9 in maintaining normal migrasome levels[10].TSPAN18 has been identified as a novel regulator of thrombo-inflammatory responses, interacting with the endothelial Orai1 calcium channel to modulate the transport, clustering, and membrane diffusion of specific interacting proteins[11].Comparison with TSPAN8, TSPAN12, TSPAN24, and TSPAN29, TSPAN27 inhibits pathological angiogenesis by regulating endothelial cell adhesion and motility[12].It has been demonstrated that migrasome formation could be dependent on specific integrin-ECM interactions, with ITGA1 and ITGA3 potentially modulating migrasome formation through the regulation of cell adhesion and migration.ITGA1/ITGB1-targeted therapies are classified for cardiovascular and cerebrovascular diseases, with elevated ITGA1 levels associated with HFpEF in type 2 diabetes and serving as both a diagnostic marker and a predictor of cardiac deterioration and readmission risk[13].In acute aortic dissection, a devastating cardiovascular disease, ITGA3 and ITGA5 are identified as key target proteins involved in the pathogenesis of the condition[14].

Recent studies have characterized four migrasome-specific marker proteins, including NDST1, PIGK, CPQ, and EOGT[15]. NDST1 is viewed as a potential target for treating chronic diseases such as hypertension and diabetes. It is involved in endothelial cell function regulation and the pathogenesis of cardiovascular diseases[16]. Defects in PIGK function may lead to neurological disorders such as neurodevelopmental disabilities, epilepsy, and cerebellar atrophy, which could indirectly affect the normal functioning of the cardiovascular system[17]. It has been observed that CPQ levels are dysregulated in the serum of heart failure patients, and CPQ may play a role in heart failure progression by influencing cardiomyocyte apoptosis and the fibrotic process. EOGT regulates the EGF-like repeats of the Notch receptor, which has potential significance in the functionality of migratory cells, and overexpression of EOGT leads to increased O-GlcNAcylation levels, which may contribute to heart failure, cardiomyopathy, and premature mortality[18]. Quantitative mass spectrometry analysis revealed that migrasomes are enriched with a series of signaling molecules, including ROCK1, TGFB2, IL1B, PDGFD, CXCL12, WNT8A, WNT11, MYDGF, BMP1, BMP7, CXCL18, WNT5B, LEFTY1, and BMP2[9]. ROCK1 is a regulatory factor in migrasome formation, and the knockdown of ROCK1 significantly reduces the number of migrasomes produced per cell. The ROCK1 inhibitor SAR407899 markedly inhibits migrasome formation without reducing the formation of retraction fibers[19]. Genome-wide association studies have identified the CXCL12 gene as being associated with coronary artery disease and MI, and plasma levels of CXCL12 are significantly correlated with the severity and prognosis of AMI[20]. TGFB2 regulates cell migration, proliferation, and differentiation through its receptors TGFβR1 and TGFβR2, and inhibiting TGFB2 can reduce the extent of cardiac fibrosis[21]. The therapeutic monoclonal antibody Canakinumab targeting IL1B has been shown to significantly reduce high inflammation risk and stabilize cardiovascular events in patients with coronary artery disease[22]. PDGFD is an angiogenic and survival factor, and its overexpression can lead to stromal fibrosis and dilated cardiomyopathy[23]. WNT11 enhances angiogenesis and improves cardiac function through the non-canonical Wnt-PKC-JNK dependent pathway, thereby increasing myocardial survival and reducing infarct size[24]. Studies have shown that MYDGF treatment reduces infarct size, increases angiogenesis in the infarcted area, and mitigates scar formation, cardiac dilation, and contractile dysfunction[25]. In the treatment of MI, BMP7 exhibits significant cardioprotective effects; early administration of BMP7 significantly increases left ventricular ejection fraction and left ventricular fractional shortening, alleviating heart failure severity and improving cardiac function[26]. LEFTY1 alleviates cardiac fibrosis following myocardial infarction by inhibiting the p-SMAD2 and p-ERK1/2 signaling pathways[27].

References

[1] LIANG H, MA X, ZHANG Y, et al. The formation of migrasomes is initiated by the assembly of sphingomyelin synthase 2 foci at the leading edge of migrating cells [J]. Nature Cell Biology, 2023, 25(8): 1173-84.

[2] HUANG Y, ZUCKER B, ZHANG S, et al. Migrasome formation is mediated by assembly of micron-scale tetraspanin macrodomains [J]. Nature cell biology, 2019, 21(8): 991-1002.
[3] YU S, YU L. Migrasome biogenesis and functions [J]. The FEBS Journal, 2022, 289(22): 7246-54.
[4] GARCIA-MAYEA Y, MIR C, CARBALLO L, et al. TSPAN1, a novel tetraspanin member highly involved in carcinogenesis and chemoresistance [J]. Biochimica et Biophysica Acta (BBA)-Reviews on Cancer, 2022, 1877(1): 188674.
[5] ZHAO J, WU W, ZHANG W, et al. Selective expression of TSPAN2 in vascular smooth muscle is independently regulated by TGF-β1/SMAD and myocardin/serum response factor [J]. The FASEB Journal, 2017, 31(6): 2576.
[6] KWON H Y, BAJAJ J, ITO T, et al. Tetraspanin 3 is required for the development and propagation of acute myelogenous leukemia [J]. Cell stem cell, 2015, 17(2): 152-64.
[7] SUN G, CHEN J, DING Y, et al. A bioinformatics perspective on the links between tetraspanin-enriched microdomains and cardiovascular pathophysiology [J]. Frontiers in cardiovascular medicine, 2021, 8: 630471.
[8] ZHANG C, DU F-H, WANG R-X, et al. TSPAN6 reinforces the malignant progression of glioblastoma via interacting with CDK5RAP3 and regulating STAT3 signaling pathway [J]. International Journal of Biological Sciences, 2024, 20(7): 2440.
[9] JIANG D, JIANG Z, LU D, et al. Migrasomes provide regional cues for organ morphogenesis during zebrafish gastrulation [J]. Nature cell biology, 2019, 21(8): 966-77.
[10] JIAO H, JIANG D, HU X, et al. Mitocytosis, a migrasome-mediated mitochondrial quality-control process [J]. Cell, 2021, 184(11): 2896-910. e13.
[11] GAVIN R L, KOO C Z, TOMLINSON M G. Tspan18 is a novel regulator of thrombo-inflammation [J]. Medical Microbiology and Immunology, 2020, 209(4): 553-64.
[12] WEI Q, ZHANG F, RICHARDSON M M, et al. CD82 restrains pathological angiogenesis by altering lipid raft clustering and CD44 trafficking in endothelial cells [J]. Circulation, 2014, 130(17): 1493-504.
[13] SU M, HOU Y, CAI S, et al. Elevated ITGA1 levels in type 2 diabetes: implications for cardiac function impairment [J]. Diabetologia, 2024, 67(5): 850-63.
[14] XING L, XUE Y, YANG Y, et al. TMT-Based Quantitative Proteomic Analysis Identification of Integrin Alpha 3 and Integrin Alpha 5 as Novel Biomarkers in Pathogenesis of Acute Aortic Dissection [J]. BioMed Research International, 2020, 2020(1): 1068402.
[15] ZHAO X, LEI Y, ZHENG J, et al. Identification of markers for migrasome detection [J]. Cell discovery, 2019, 5(1): 27.
[16] ADHIKARI N, BASI D L, TOWNSEND D, et al. Heparan sulfate Ndst1 regulates vascular smooth muscle cell proliferation, vessel size and vascular remodeling [J]. Journal of molecular and cellular cardiology, 2010, 49(2): 287-93.
[17] NGUYEN T T M, MURAKAMI Y, MOBILIO S, et al. Bi-allelic variants in the GPI transamidase subunit PIGK cause a neurodevelopmental syndrome with hypotonia, cerebellar atrophy, and epilepsy [J]. The American Journal of Human Genetics, 2020, 106(4): 484-95.
[18] ALAM S M D, TSUKAMOTO Y, OGAWA M, et al. N-Glycans on EGF domain-specific O-GlcNAc transferase (EOGT) facilitate EOGT maturation and peripheral endoplasmic reticulum localization [J]. Journal of Biological Chemistry, 2020, 295(25): 8560-74.
[19] LU P, LIU R, LU D, et al. Chemical screening identifies ROCK1 as a regulator of migrasome formation [J]. Cell discovery, 2020, 6(1): 51.
[20] ZHANG C, LI T, YIN S, et al. Monocytes deposit migrasomes to promote embryonic angiogenesis [J]. Nature Cell Biology, 2022, 24(12): 1726-38.
[21] FRANGOGIANNIS N G. Transforming growth factor-β in myocardial disease [J]. Nature Reviews Cardiology, 2022, 19(7): 435-55.
[22] GOOD E, DE MUINCK E. Targeting systemic inflammation in atherosclerosis: Who will benefit? [Z]. SAGE Publications Sage UK: London, England. 2018: 921-2
[23] KIM H-J, CHENG P, TRAVISANO S, et al. Molecular mechanisms of coronary artery disease risk at the PDGFD locus [J]. Nature communications, 2023, 14(1): 847.
[24] WANG J, GONG M, ZUO S, et al. WNT11-conditioned medium promotes angiogenesis through the activation of non-canonical WNT-PKC-JNK signaling pathway [J]. Genes, 2020, 11(11): 1277.
[25] KORF-KLINGEBIEL M, REBOLL M R, KLEDE S, et al. Myeloid-derived growth factor (C19orf10) mediates cardiac repair following myocardial infarction [J]. Nature medicine, 2015, 21(2): 140-9.

[26] MERINO D, VILLAR A V, GARCíA R, et al. BMP-7 attenuates left ventricular remodelling under pressure overload and facilitates reverse remodelling and functional recovery [J]. Cardiovascular research, 2016, 110(3): 331-45.
[27] LI C-Y, ZHANG J-R, LI X-X, et al. Lefty1 ameliorates post-infarction fibrosis by suppressing p-Smad2 and p-ERK1/2 signaling pathways [J]. Journal of cardiovascular translational research, 2021, 14: 636-46.

Literature review of reported AMI signatures

We collected 63 published AMI signatures. Detailed information, including signature genes and references, can be found in Supplementary Table 2. Below, we mainly summarized the approaches and results of these signatures:

Model 1: Evan D. Muse et al. used the Differentially Expressed Genes (DEGs) Receiver Operating Characteristic (ROC) analyses to identify a whole blood circulating endothelial cells (CEC)-derived molecular signature, which was composed of 11 genes including HBEGF, SYTL3, EDN1, NR4A2, NFKBIA, VPS8, NR4A3, SULF1, RNASE1, CCL20, and MGP.

Model 2: Nan Zhang et al. combined the Univariate Regression and Least Absolute Shrinkage and Selection Operator (LASSO) to identify an 11 immune gene signature (ADAMTS1, CNN2, DHRS13, DUSP1, FASLG, GNPTAB, NARF, PHC2, RAB7A, VNN3, and YIPF3).

Model 3: Yanze Wu et al. constructed a gene signature-based predictive model by Combining Random Forest (RF) and Artificial Neural Network (ANN), which was composed of 11 genes including (ITLN1, NFIL3, ZFP36, MCEMP1, ACSL1, BCL6, IRAK3, IL1R2, SERPINA1, DYSF, and S100A12).

Model 4: Haihua Pan et al. integrated the Stepglm and Enet algorithms to develop a gene signature in early acute myocardial infarction patients (CLEC2D, TCN2, and CCR1).

Model 5: Ming Li et al. integrated Monte Carlo feature selection (MCFS), incremental feature selection (IFS), and support vector machine (SVM) to identify a signature composed of DLGAP1-AS1, PYGL, MEGF9, PHC2, IL1R1, TLR2, and TLR4.

Model 6: Jason J Rose et al. performed a logistic regression to establish a signature composed of GNB1, CALU, PRDX6, PPP2R1A, PSAP, SPARC, ACTR2, ZYX, and GAS6.

Model 7: Meng Xia et al. built a protein-protein interaction network using differentially expressed Tregs-related genes (DETregRGs), identifying PTPRC, C3AR1, CD53, TLR2, and CCR1.

Model 8: Hiromi W L Koh et al. used a network-based data integration approach (iOmicsPASS+) and LASSO to generate an integrated signature of extracellular matrix proteins composed of EFEMP1 and FSTL3.

Model 9: Jing Xu et al. performed the least absolute shrinkage and selection operator (LASSO) to identify eight genes, including ITGAM, CLEC4D, SLC2A3, BST1, MCEMP1, PLAUR, GPR97, and MMP25.

Model 10: Siyu Guo et al. established a PPI approach to identify a ten-gene signature composed of CXCL8, TNF, FPR2, CXCL1, JUN, IL1B, PPBP, MMP9, TLR2, and FCER1G.

Model 11: Yanze Wu et al. established a PPI approach to identify a five-gene signature composed of TLR2, IL1B, LILRB2, FCER1G, FPR1, and MMP9.

Model 12: Jin Zhou et al. established a PPI approach to identify a three-gene signature composed of BCL6, PTGS2, and PTEN.

Model 13: Junqiang Xue et al. used WGCNA to identify a gene signature composed of RPL9, RPL26, OLR1, FOS, BCL6, TLR2, IRS2, IER3, TNFAIP6, B4GALT5, CEBPD, NFIL3, TRIB1, PTGS2, MXD1, BCL3, and PLAUR.

Model 14: Rui Zhang et al. established a PPI approach to identify a gene signature composed of ARG1, PLAUR, FOS, and IL1R2.

Model 15: Dongsheng Wei et al. conducted DEGs, ROC analysis, and experiment validation to propose NR4A2 as a diagnostic biomarker.

Model 16: Jiahe Wu et al. established a PPI approach on ferroptosis-related genes (FRGs) to identify a seven gene signature composed of ALOX5, CAMKK2, KDM6B, LAMP2, PTEN, PTGS2, and ULK1.

Model 17: Yun Xie et al. used DEGs and ROC analyses to identify a four gene signature composed of FN1, CD34, LPL, and WWTR1.

Model 18: Qixin Chen et al. combined DEGs, WGCNA, and ROC analyses to PRKAR1A and SDCBP as diagnostic biomarkers.

Model 19: Liting Yang et al. performed an SVM algorithm to identify a signature including ACOX1, BCL6, CEACAM8, CUGBP2, and GPX7.

Model 20: Jie Xiang et al. established a PPI approach based on senescence-related genes to identify a three gene signature composed of MMP9, ETS2, and BCL6.

Model 21: Zheng Liu et al. used DEGs and ROC analyses based on cuproptosis-related genes (CRGs) to screen six genes, including LIAS, LIPT1, DLAT, PDHB, MTF1, and GLS.

Model 22: Jiahe Wu et al. established a PPI approach to identify a three-gene signature composed of GPM6A, SRSF1, and ANK2.

Model 23: Kai Liu et al. integrated WGCNA and PPI to screen a ten-gene signature, including Atf3, Ptgs2, Cxcl1, Socs3, Hspa1b, Selp, Cxcl2, Il1b, Myd88, and S100a8.

Model 24: Guochang Zhang et al. combined the DEGs analysis and miRNA-mRNA-protein regulatory network to identify KLRC4, KLRC2, and DLC1.

Model 25: Yongwei Yu et al. performed DEGs analysis to identify ten genes, including Il6, Spp1, Ptgs2, Serpine1, Plaur, Cxcl5, Lgals3, Serpinb2, and Cd14.

Model 26: Jijuan Wang et al. used DEGs analysis based on sphingolipid metabolism-related genes to screen out six genes (Asah1, Degs1, Neu1, Sptlc2, Sphk1, and Gba2) that were significantly associated with AMI.

Model 27: Yonghao Jiang et al. used WGCNA and DEGs analysis on ferroptosis-related genes to identify a five-gene signature, including ATM, PIK3CA, MAPK8, KRAS and SIRT1.

Model 28: Nai Zhang et al. established a PPI approach to identify a three-gene signature composed of CEP55, NMU, CAV1, SEMA6A, TBX3, FBLN1, and SYNM.

Model 29: Yuan Gao et al. established a PPI approach to identify a six-gene signature composed of CCL5, BCL3, NR2C2, MAX, GTF3C2, and NCOA7.

Model 30: Hongjun You et al. constructed an ANN based on the differentially expressed immuno-inflammation-related genes (DEIIRGs) with diagnostic performance, including SH2D1B, ADM, PI3, MMP9, NRG1, CBLB, RORA, and FASLG.

Model 31: Xianpei Wang et al. identified IGF2BP1, FTO, RBM15, METTL3, YTHDC2, FMR1, and HNRNPA2B1 as the seven major m6A regulators via DEGs analysis.

Model 32: Sanjay Kumar et al. used DEGs and WGCNA approaches to identify four genes, including ADOR-A3, BMP6, VPS8, and GPx3.

Model 33: Shuo Feng et al. used the PPI approach to identify a six-gene signature composed of MMP9, ARG1, CA4, CRISPLD2, S100A12, and GZMK.

Model 34: Xiang Chen et al. leveraged the LASSO to identify SYTL2, KLRD1, and C12orf75 as potential diagnostic biomarkers from circulating cells.

Model 35: Jingqi Yang et al. performed the LASSO and RF on programmed cell death-related genes and identified key genes, including ASB13 and CDCA7.

Model 36: Nader Ebadi et al. used the DEGs analysis to identify the top three upregulated genes, including KDM5D, EIF1AY, and CCL20.

Model 37: Shengjue Xiao et al. integrated the DEGs analysis and WGCNA to identify FCER1G and PTGS2 as diagnostic biomarkers.

Model 38: Yajuan Du et al. performed the support vector machine-recursive feature elimination (SVM-RFE) based on autophagy-related genes (ARGs) and identified a seven gene signature, which was composed of WDFY3, TP53INP2, GABARAPL1, CDKN1A, DDIT3, NAMPT, and FOS.

Model 39: Kun Jiao et al. used the PPI approach identify S100A9, mitogen-activated protein kinase (MAPK) 3, MAPK1, MMP3, interleukin (IL)-17A, and HSP90AB1 as hub gene sets.

Model 40: Siyu Guo et al. used the DEGs and PPI analyses to identify a ten-gene panel, including BDKRB1, BDKRB2, CCL25, HRH1, KISS1, NPBWR1, GRPR, TACR3, HTR2B, and ORM1.

Model 41: Hu Zhai et al. used the DEGs and PPI analyses to identify a five- gene panel, including IL2RB, IL18RAP, PROK2, S100A12, VNN2.

Model 42: Cheng Yu et al. used the PPI approach to identify a six-gene signature composed of TLR2, HP,

ICAM1, LCN2, LTF, VCAN, S100A9 and NFKBIA.

Model 43: Daqiu Chen et al. used the PPI approach and identified the most fundamental genes, including CCR1, CXCL1, CXCL2, CXCLl5, and MMP8.

Model 44: S-J Xiao et al. used the DEGs and PPI approaches to identify a six-gene signature composed of PTAFR, AQP9, TLR4, HCAR3, LRG1, and SMAD4.

Model 45: Xuefei Li et al. performed a time-series DEGs analysis, followed by an network analysis of PPI, and identified four downregulated genes, including FADS2, LRRN3, GPR15, and AK5.

Model 46: Abdulrahman Mujalli et al. integrated DEGs, PPI, and gene network-clusters to screen IL1B, IL1A, IL1RN, STAT3, PTX3, PTGS2, and JUN as diagnostic biomarkers.

Model 47: Yi Yang et al. used the DEGs and PPI approaches and identified two hub genes, SRC and FYN.

Model 48: Tong Zhang et al. performed a time-series DEGs analysis and identified MMP9, ITGB2, ICAM1, and MLYCD as hub genes.

Model 49: Hao Li et al. used the PPI approach and Fuzzy C-Means Clustering (FCM) to identify a six-gene signature composed of MYL7, TSC22D2, HSPA1A, BTG2, NR4A1, and RYR2.

Model 50: Lingxiao Wang et al. identified ten strongly interlinked hub genes (Timp1, Sparc, Spp1, Tgfb1, Decr1, Vim, Serpine1, Serpina3n, Thbs2, and Vcan) by PPI.

Model 51: Yao Xie et al. identified differentially expressed proteins (DEPs) of AMI patients, in which PLG, C8B, and F2 were selected as candidate molecules.

Model 52: Ke Wu et al. used the PPI approach to identify a gene signature, which was composed of TFEB, IRS2, GRB2, FASLG, LIMS1, CX3CR1, HSPA6, TPM3, LAT2, CEBPD, AQP9, and MAPKAPK2.

Model 53: Qiaoyu Zhou et al. used DEGs and WGCNA, and screened out four hub genes (CSF2RB, SIGLEC9, LRRC25, and CSF3R) to be differentially expressed and to have high diagnostic value.

Model 54: Yushuang Yang et al. identified three genes, including STAT3, LCK, and FYN, from the PPI network and/or the transcriptional regulatory network.

Model 55: Shan Wang et al. identified the five hub genes with the highest connection scores, including ITGAM, CD163, ARG1, HIF1A, and ACSL1, through PPI network analysis.

Model 56: Yiqun Guo et al. used LASSO and SVM-RFE algorithms to identify hub 5mC regulators, including DNMT3B, MBD3, UHRF1, UHRF2, NTHL1, SMUG1, ZBTB33, TET1, and TET3.

Model 57: Guangyao Shao et al. used the DEGs and PPI network analyses and identified AQP9, IL1B, and IL1RN as potential immune-related biomarkers.

Model 58: Zhengyu Liu et al. perfumed LASSO and SVM-RFE screening, and four genes (ACSL1, CH25H, GPCPD1, and PLA2G12A) were identified as potential diagnostic biomarkers.

Model 59: Qixin Chen et al. used the DEGs and WGCNA analyses, and identified CUX1, CTSD and ADD3

as potential biomarkers.

Model 60: Hongyu Li et al. combined the SVM-REF, LASSO, and RF screening, and obtained the overlapping genes including ACSL1, GABARAPL1, IL1R2, IRAK3, MCEMP1, NFIL3, and THBD.

Model 61: Wei Xiong et al. conducted DEGs analysis and identified two hub genes (IGLON5 and LMX1A).

Model 62: Pengfei Zheng et al. performed WGCNA and identified CDC42, JAK2, and CHUK with the top three-degree values.

Model 63: Traditional AMI biomarkers include TNNT2, TNNI3, and MB.
