# Supplementary material for: Unveiling the Potential of Migrasomes: A Machine-Learning-Driven Signature for Diagnosing Acute Myocardial Infarction
Source: Biomedicines. 2024 Jul 22;12(7):1626. doi: 10.3390/biomedicines12071626 (PMC11274667; doi:10.3390/biomedicines12071626)
Supplement: Supplementary file 1 [file biomedicines-12-01626-s001.zip › biomedicines-3087802-supplementary/Supplementary File S2.pdf]

Supplementary description of method

Development and validation of migrasome-related signature (MS)

To develop a MS with high accuracy and stability performance, we integrated 12 machine learning algorithms including least absolute shrinkage and selection operator (Lasso), ridge, elastic network (Enet), stepwise generalized linear model (Stepglm), support vector machine (SVM), gradient boosting with component-wise linear models (glmBoost), linear discriminant analysis (LDA), partial least squares regression for generalized linear models (plsRglm), random forest (RF), gradient boosting machine (GBM), eXtreme gradient boosting (XGBoost), and NaiveBayes.

(1) Lasso, Ridge, and Enet

To address the overfitting that occurs in linear regression, the Lasso, Ridge, and Enet models merged as the most popular approaches to regularized linear regression, which were developed. Lasso introduces the L1 penalty, which can realize feature screening and alleviate the overfitting problem. Ridge adds the L2 penalty, which can solve the covariance problem, but does not have the ability to screen feature. Enet is a combination of Lasso and Ridge, which adds both L1 and L2 penalties on the basis of linear regression, and has the functions of feature screening and covariance solvent.

Experimental setting: The Lasso, ridge, and Enet models were implemented using the R package glmnet. The optimal regularization parameter, lambda, was determined by 10-fold cross-validation, whereas the L1-L2 parameter, α, was set to 0-1 (interval =0.1).

(2) Stepglm

Stepglm constructs optimal generalized linear models by automated stepwise selection, usually based on AIC or BIC. Stepglm performs feature selection and is suitable for building a series of multiple regression models that require step-by-step screening of important features.

Experimental setting: We used the R package glm to construct the Stepglm model under AIC. The directions were respectively set as both, forward, and backward.

(3) SVM

SVM is a supervised learning model, especially suited for classification problems.

SVM performs classification by finding a hyperplane in a high-dimensional space that maximizes the boundary. Nonlinear problems can be handled using kernel functions. Experimental setting: The SVM model was implemented via the R package e1071. The SVM-Kernel and cost were set as radial and 1, respectively.

(4) glmBoost

As a generalized linear model fitted on a boosting approach based on component-wise univariate linear models, glmBoost was established by fitting the residuals stepwise, which exploits the power of the boosting algorithm and the flexibility of generalized linear models. Gradient boosting for optimizing arbitrary loss functions where component-wise linear models are utilized as base-learners.

Experimental setting: The glmBoost model was implemented via the R package mboost. The cvrisk function used the 10-fold cross-validation to estimate the empirical risk for hyper-parameter selection.

(5) LDA

LDA is a linear classification model that performs classification by finding linear combinations that maximize the between-class variance and minimize the within-class variance.

Experimental setting: The LDA model was implemented via the R package MASS. The CV parament was set as TRUE, which returns the classes and posterior probabilities for 10-fold cross-validation.

(6) plsRglm

Considering the covariance of response variables and characteristic variables, plsRglm combines partial least squares regression and generalized linear models for regression analysis, which is suitable for high-dimensional datasets with severe multicollinearity. Experimental setting: The plsRglm was implemented via the R package plsRglm. We used the cv.plsRglm function to establish the model with 10-fold cross-validation. The modele parameter was set as pls-glm-logistic for classification.

(7) RF

RF is an ensemble learning method that performs classification or regression by constructing multiple decision trees and combining their predictions. RF could improve

the generalization ability through randomly selecting features and data subsets. Experimental setting: The RF model was implemented via the R package randomForestSRC. The function tune.rfsrc was used to tune the optimal mtry and nodesize. We used a grid-search on ntree and mtry using 10-fold cross-validation. All the pairs of ntree and mtry were formed and the one with the best AUC value was identified as the optimized parameters.

(8) GBM

GBM is a boosting method that constructs multiple weak learners, usually decision trees, by fitting residuals step by step and combining them into one strong learner. Each step attempts to correct the errors of the previous step.

Experimental setting: The GBM model was implemented via the R package gbm. Using the 10-fold cross-validation, the cv.gbm function selected index for number trees with minimum cross-validation error. The gbm function was used to fit the generalized boosted regression model.

(9) XGBoost

XGBoost is an efficient implementation of GBM that employs parallel computing, tree structure optimization, and regularization techniques to greatly improve the performance and speed of the model.

Experimental setting: The XGBoost model was implemented via the R package xgboost. To achieve a tree-based XGBoost with minimum cross-validation error, we used the 10-fold cross validation to select the optimal booster parameters.

(10) NaiveBayes

NaiveBayes is a classification model that assumes features are independent of each other, which performs classification by computing the conditional a-posterior probabilities of a categorical class variable given independent predictor variables using the Bayes rule.

Experimental setting: The NaiveBayes model was implemented via the R package e1071. The parameters were set as default.
