# Supplementary figures and images for: Unveiling the Potential of Migrasomes: A Machine-Learning-Driven Signature for Diagnosing Acute Myocardial Infarction
Source: Biomedicines. 2024 Jul 22;12(7):1626. doi: 10.3390/biomedicines12071626 (PMC11274667; doi:10.3390/biomedicines12071626)

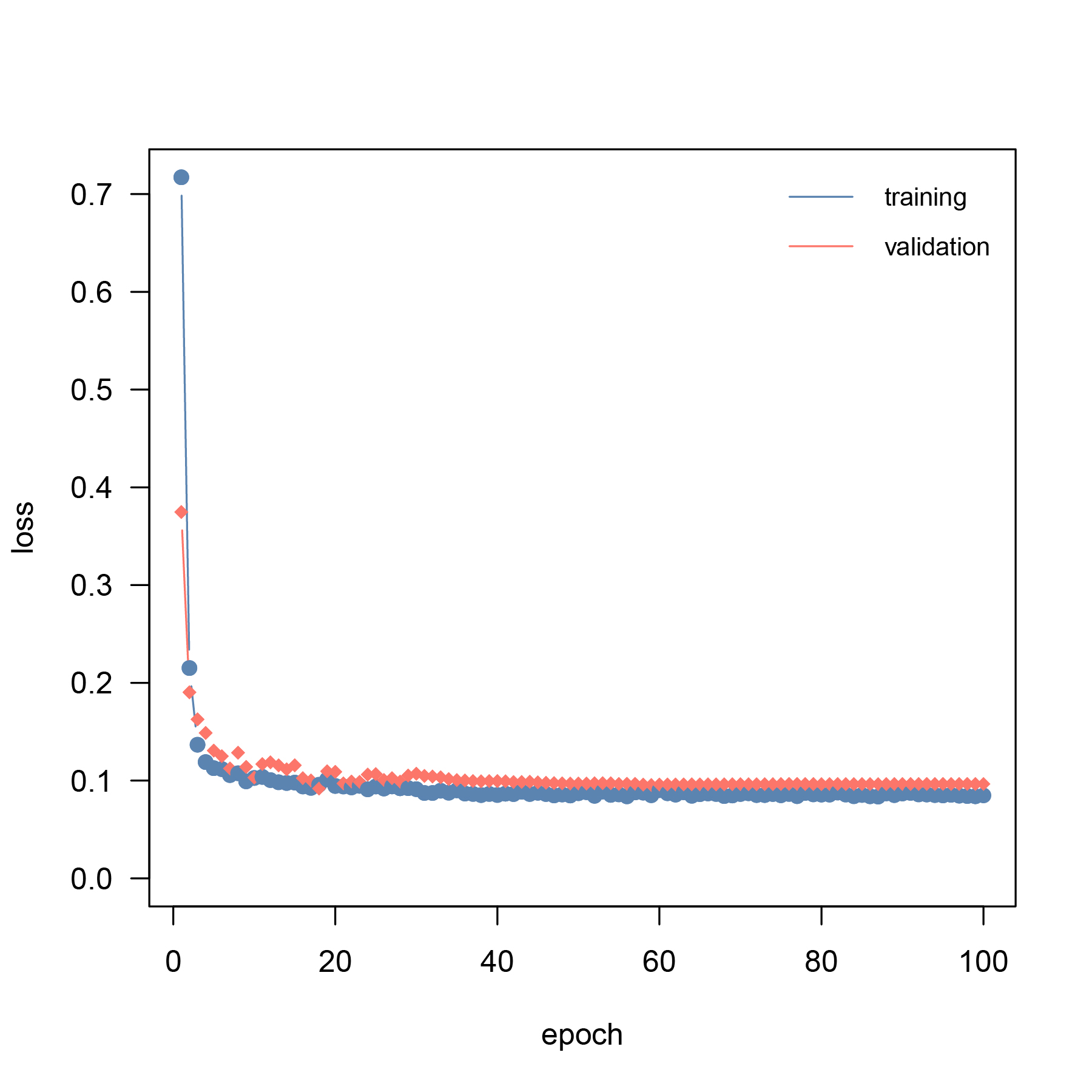

Supplement: Supplementary file 1 [file biomedicines-12-01626-s001.zip › biomedicines-3087802-supplementary/Supplementary Figure S1.jpg]

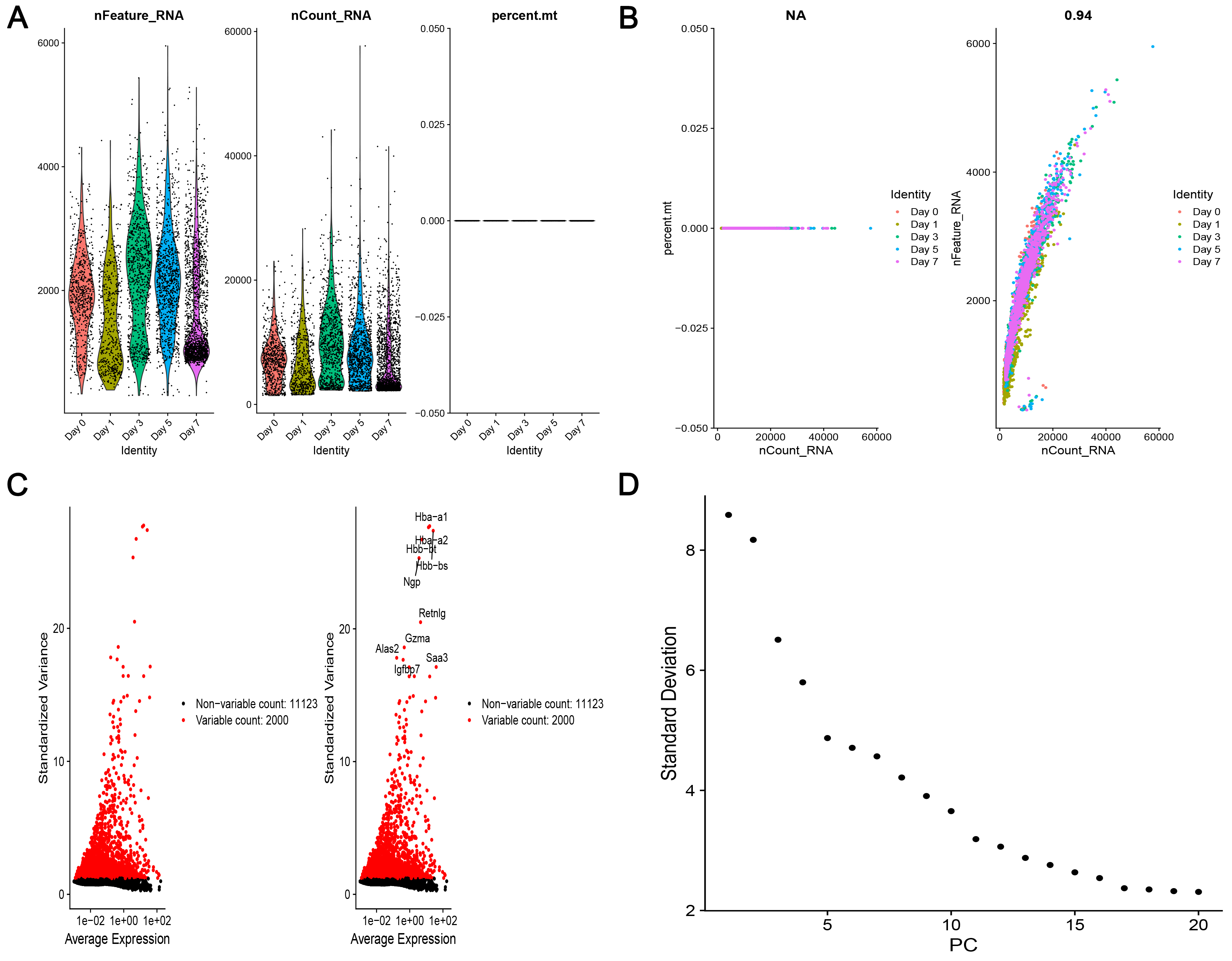

Supplement: Supplementary file 1 [file biomedicines-12-01626-s001.zip › biomedicines-3087802-supplementary/Supplementary Figure S2.jpg]

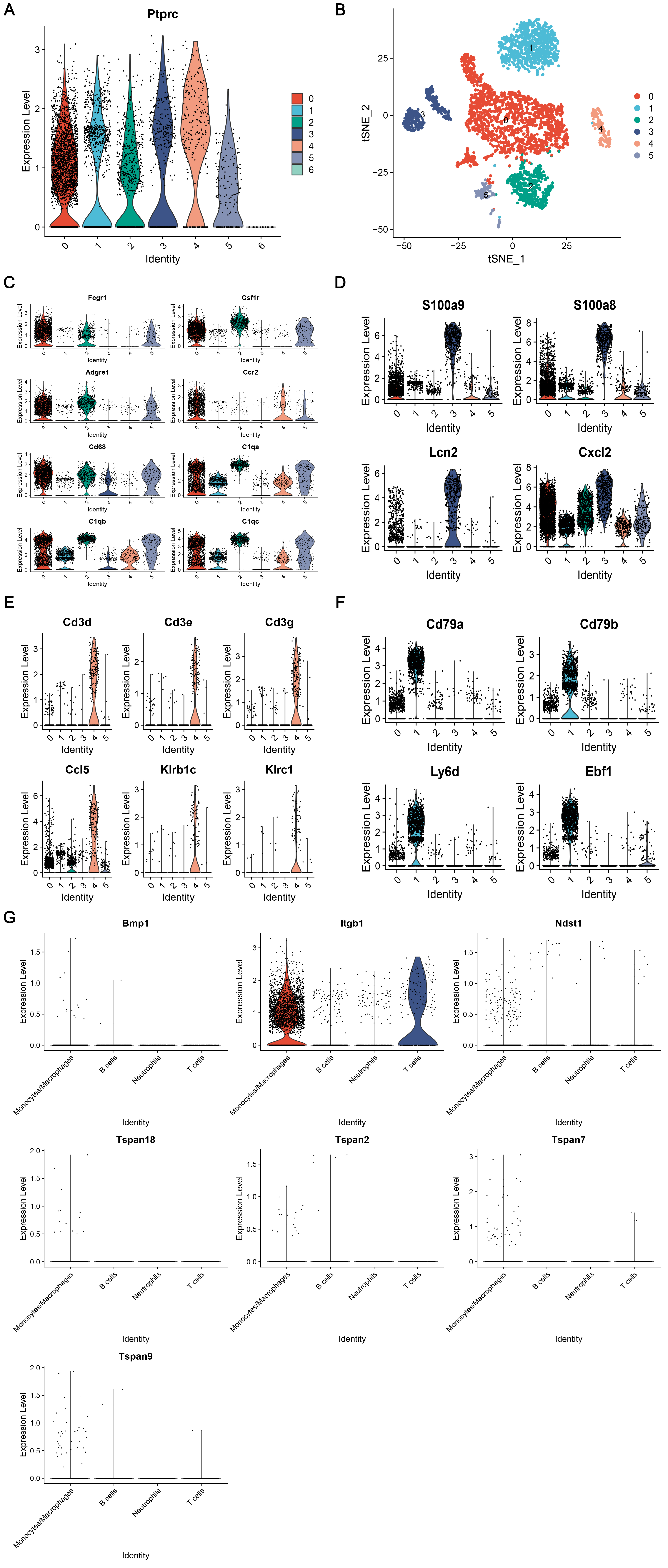

Supplement: Supplementary file 1 [file biomedicines-12-01626-s001.zip › biomedicines-3087802-supplementary/Supplementary Figure S3.jpg]

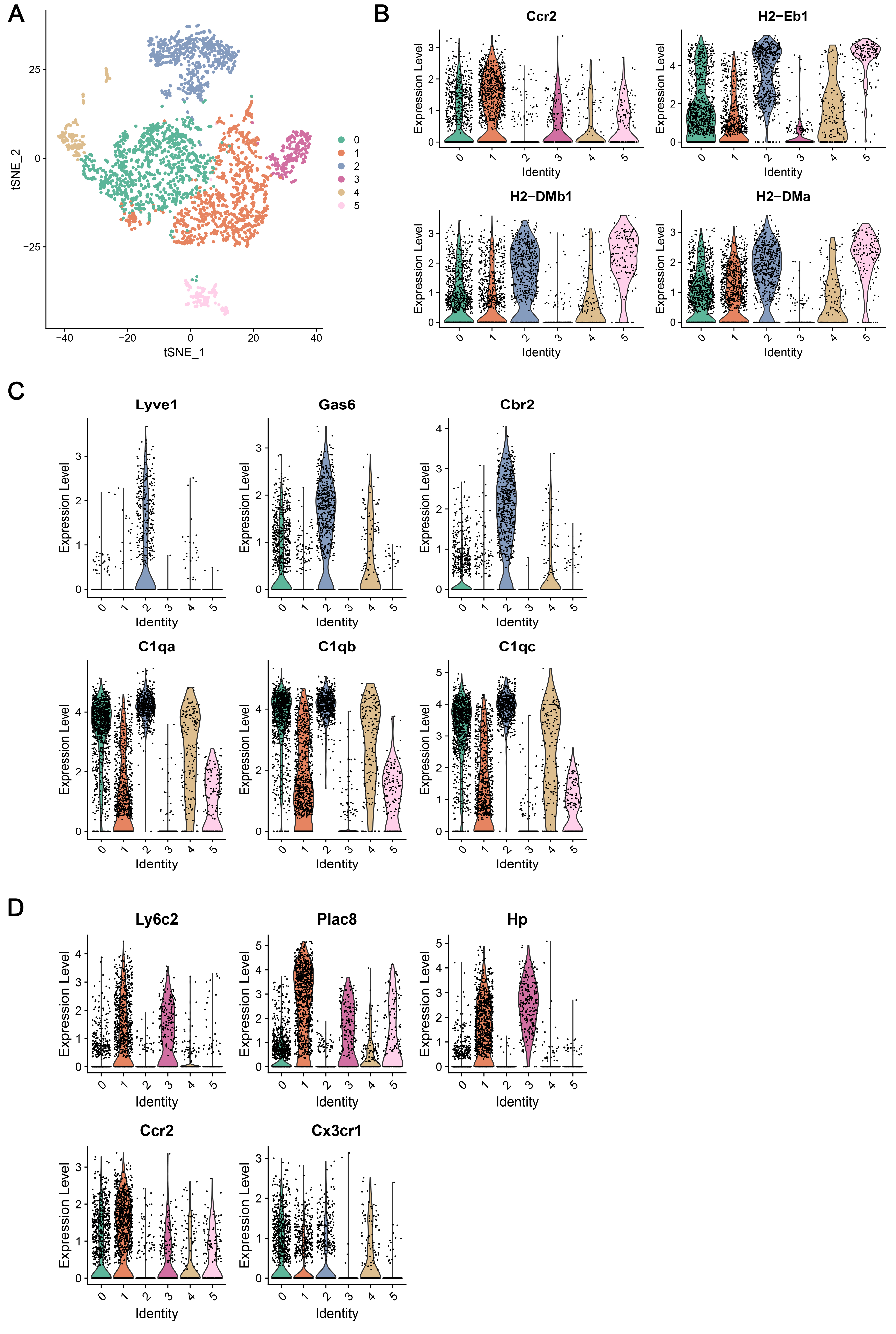

Supplement: Supplementary file 1 [file biomedicines-12-01626-s001.zip › biomedicines-3087802-supplementary/Supplementary Figure S4.jpg]
